# Sub-MIC antibiotics increased the fitness cost of CRISPR-Cas in *Acinetobacter baumannii*

**DOI:** 10.3389/fmicb.2024.1381749

**Published:** 2024-07-01

**Authors:** Ting Yu, Jiayuan Huang, Xinyue Huang, Jingchen Hao, Pengyu Zhang, Tingting Guo, Guangyu Bao, Guocai Li

**Affiliations:** ^1^Department of Microbiology, Institute of Translational Medicine, Medical College, Yangzhou University, Yangzhou, China; ^2^Department of Laboratory Medicine, Affiliated Hospital, Yangzhou University, Yangzhou, China; ^3^Jiangsu Key Laboratory of Zoonosis/Jiangsu Co-Innovation Center for Prevention and Control of Important Animal Infectious Diseases and Zoonoses, Yangzhou University, Yangzhou, China; ^4^Jiangsu Key Laboratory of Integrated Traditional Chinese and Western Medicine for Prevention and Treatment of Senile Diseases, Medical College/Guangling College, Yangzhou University, Yangzhou, China

**Keywords:** *Acinetobacter baumannii*, CRISPR-Cas, sub-MIC, fitness cost, metabolomics

## Abstract

**Introduction:**

The escalating prevalence of bacterial resistance, particularly multidrug-resistant bacteria like *Acinetobacter baumannii*, has become a significant global public health concern. The CRISPR-Cas system, a crucial defense mechanism in bacteria against foreign genetic elements, provides a competitive advantage. Type I-Fb and Type I-Fa are two subtypes of CRISPR-Cas systems that were found in A. baumannii, and the I-Fb CRISPR-Cas system regulates antibiotic resistance in *A. baumannii*. However, it is noteworthy that a majority of clinical isolates of *A. baumannii* lack or have incomplete CRISPR-Cas systems and most of them are multidrug-resistant. In light of this, our study aimed to examine the impact of antibiotic pressure on the fitness cost of the I-Fb CRISPR-Cas system in *A. baumannii*.

**Methods and Results:**

In the study, we conducted in vitro competition experiments to investigate the influence of sub-minimum inhibitory concentration (sub-MIC) on the CRISPR-Cas systems’ fitness cost in *A. baumannii*. We found that the fitness cost of the CRISPR-Cas system was increased under sub-MIC conditions. The expression of CRISPR-Cas-related genes was decreased, while the conjugation frequency was increased in AB43 under sub-MIC conditions. Through metabolomic analysis, we identified that sub-MIC conditions primarily affected energy metabolism pathways. In particular, we observed increased carbon metabolism, nitrogen metabolism, and intracellular ATP. Notably, the CRISPR-Cas system demonstrated resistance to the efflux pump-mediated resistance. Furthermore, the expression of efflux pump-related genes was increased under sub-MIC conditions.

**Conclusion:**

Our findings suggest that the I-Fb CRISPR-Cas system confers a significant competitive advantage in *A. baumanni*. However, under sub-MIC conditions, its function and the ability to inhibit the energy required for efflux pumps are reduced, resulting in an increased fitness cost and loss of competitive advantage.

## Introduction

Due to its robust survival capabilities, *A. baumannii*, a non-fermenting Gram-negative bacterium (De Silva and Kumar, 2019; Muller et al., 2023) has become the most common source of hospital-acquired infections such as ventilator-associated pneumonia, bloodstream infections, and urinary tract infections (Guo et al., 2022; Cavallo et al., 2023). Over the years, owing to the widespread use of antibiotics, the resistance rate of *A. baumannii* is continually increasing (Peleg et al., 2008; Castillo-Ramirez, 2023), making it one of the most critical bacteria for which new antibiotics are urgently needed, as declared by the World Health Organization (Boucher et al., 2009; Tacconelli et al., 2018).

Currently, the clinical management of *A. baumannii* primarily relies on antibiotics. However, with the rising rates of antibiotic resistance, there is an urgent need to explore alternative approaches to prevent resistance. The clustered regularly interspaced short palindromic repeats (CRISPR) and CRISPR-associated protein (CRISPR-Cas) system is a remarkable immune system in prokaryotes that provides adaptive immunity against foreign genetic elements (Kao et al., 2021; Mortensen et al., 2021), such as bacteriophages and plasmids (Li et al., 2016; Guo et al., 2022). The CRISPR-Cas system has been classified into two categories, with six types (I-VI) and over 30 subtypes based on the composition of effector complexes (Huang and Zhu, 2020). In *A. baumannii*, the most prevalent CRISPR-Cas system type is the I-F subtype (Guo et al., 2022; Wang et al., 2022). The I-Fb CRISPR-Cas system consists of Cas1, Cas3, Csy1, Csy2, Csy3, Csy4, and CRISPR arrays (Wang et al., 2022; Camara-Wilpert et al., 2023). The CRISPR RNA, which is transcripted from CRISPR arrays, together with Cas proteins (Csy1–4) form an interference complex to recognize and target specific sequences (Sinkunas et al., 2011; Luo et al., 2015). Cas1 plays a crucial role in integrating the spacer into the CRISPR site, while Cas3 cleaves invading RNA (Sinkunas et al., 2011; Luo et al., 2015).

In addition to immune function, the CRISPR-Cas systems in bacteria also play a role in inhibiting the horizontal transfer of resistance genes (Westra and Levin, 2020). Pursey et al. (1842), Palmer and Gilmore (2010), and Tong et al. (2017) found that the strains possessing the CRISPR-Cas system were less likely to carry the resistance genes. Guo et al. (2022) found that the *A. baumannii* with a complete CRISPR-Cas system was susceptible despite resistance genes. Indeed, the CRISPR-Cas system can inhibit the expression of resistance genes, thereby preserving the sensitivity to antibiotics. Sensitive strains have lower fitness costs and competitive advantage without antibiotic pressure (Wen et al., 2021), indicating their better survival in an antibiotic-free environment. In addition, Wang et al. (2022) found that the CRISPR-Cas systems in *A. baumannii* are primarily incomplete, raising the question: why does this loss occur in *A. baumannii*? Antibiotics are drugs used to treat or inhibit microbial infections, but their widespread use has led to human and environmental exposure to residual antibiotics (Carlet et al., 2012; Ma et al., 2017; Mehanni et al., 2023). Under the drug pressure, acquiring resistance genes has become a necessary survival strategy for bacteria (Westra and Levin, 2020). The CRISPR-Cas system, which can inhibit the horizontal transfer of resistance genes, presents a hurdle for bacteria in acquiring resistance genes (Pawluk et al., 2018). To acquire resistance genes for survival, the CRISPR-Cas system may gradually be suppressed and lost. As a result, the mutants lacking the CRISPR-Cas system may acquire resistance genes and become dominant populations (Wang et al., 2022). Thus, it is reasonable to speculate that the presence of sub-MIC in the environment increases the fitness cost for bacteria possessing the CRISPR-Cas system, which in turn may impact the existence of the CRISPR-Cas system.

In the present study, we test the hypothesis that the drug pressure increases the fitness cost of bacteria with intact CRISPR-Cas systems, which in turn results in the loss of CRISPR-Cas systems in these bacteria.

## Materials and methods

### Bacterial strains

The strain AB43 (GenBank: CP083182.1,^1^) carrying a complete I-Fb CRISPR-Cas system, was isolated from the Affiliated Hospital of Yangzhou University, and the mutant AB43ΔCRISPR-Cas, lacking the completed CRISPR-Cas system in AB43, was constructed in our laboratory (Wang et al., 2022). The strain AB43-RR (rifampicin resistance) was derived from AB43 through continuous subculture under rifampicin (RIF) (IR0110, Solarbio, Beijing, China) concentration gradient. Briefly, AB43 was cultured in Luria-Bertani (LB) broth (HB0128, Hopebio, Qingdao, China), containing 1 μg/mL RIF for 18 h, and then subcultured in another LB broth containing a 2-fold increasing RIF concentration with 1% inoculum until the final concentration of RIF reached 200 μg/mL. The AB219, AB227, AB300, and Aby1 were obtained from the Affiliated Hospital of Yangzhou University. The subcultures were stored with 20% glycerol in a −80°C freezer for future use.

### Growth rates and determination of MIC

The MICs of the strains were measured by the microdilution method according to the Clinical and Laboratory Standards Institute (CLSI, 2024). The MICs for the Ceftriaxone (CRO) (C7780, Solarbio, Beijing, China), Levofloxacin (LFX) (L8730, Solarbio, Beijing, China), and Tetracycline (TET) (T8180, Solarbio, Beijing, China), were performed by the 2-fold broth dilution in 96-well culture plates (Corning, USA). Briefly, the bacterial inoculum was diluted in Mueller-Hinton (MH) broth (HB6231, Hopebio, Qingdao, China) to a concentration of 0.5 × 10^6^ CFU/mL, and 100 μL of the diluted inoculum was added to a 96-well of the plate (Yilmaz et al., 2020) and the respective test antibiotics, prepared from 2-fold serial dilutions in MH broth. The plates were incubated for 18 h at 37°C, and the minimum concentration of antibiotic without visible bacterial growth was recorded as MIC.

Growth curves of strains were measured at sub-MIC concentrations (Jorba et al., 2021). Exponential-phase cultures of the strains were cultured in LB broth and then diluted to OD_600_ = 0.5 using LB broth. These cultures were then inoculated at a volumetric proportion of 1% in a final volume of 15 mL LB broth, supplemented with antimicrobial (CRO, LFX, and TET) at a concentration of 1/4 MIC and 1/2 MIC. RTS-1C real-time cell growth loggers (Biosan SIA, Riga, Latvia) were used to monitor growth. The cultures were incubated for 24 h at 37°C with shaking at 1500 rpm. Growth was measured every 30 min by recording the optical density at a wavelength of 850 nm. The OD_600_ was calculated using the formula OD_600_ = OD_850_ × 1.9.

### *In vitro* competition assays

*In vitro* competition experiments were measured as described in previous studies (Yang et al., 2017; Wen et al., 2021). 1/4 and 1/2 MIC of antibiotics were used in the current *in vitro* competition assays based on the bacteria growth curves with various antibiotic concentrations. The AB43 and AB43ΔCRISPR-Cas strains were cultured in LB broth and diluted to OD_600_ = 0.5 using LB broth and then mixed at volume ratios of 1:2, 1:1, and 2:1, respectively. Each mixture was added to 5 mL of LB broth supplemented with the 1/4 and 1/2 MIC antibiotics, with an initial inoculation of 1%. Samples at 0, 24, 48, and 72 h were serially 10-fold diluted and spotted onto LB agar plates and antibiotic agar plates, respectively, to calculate the colony-forming units (CFU). The ratio of AB43ΔCRISPR-Cas (CFU in antibiotic agar plates) is calculated as the number of AB43ΔCRISPR-Cas multiplied by 100% divided by the sum of the number of AB43 and AB43ΔCRISPR-Cas (CFU in LB agar plates).

### Detection of the carbon, nitrogen, and energy metabolism in *Acinetobacter baumannii*

To assess the carbon source utilization of the strains, we employed the Biolog-ECO technology as described in previous studies (Yang et al., 2019; Wen et al., 2021), which allowed us to measure the metabolism of 31 different carbon sources. First, the strains were cultured at 37°C and then diluted to OD_600_ = 0.5 using LB broth. The strains were washed three times with an equal volume of 1 x phosphate-buffered saline (PBS). Subsequently, samples were diluted 1:1000 with saline to obtain a sample dilution. A total of 150 μL of the bacterial dilution was added to each well of an ECO MicroPlate (BIOLOG, USA). The plates were then incubated at 25°C in the dark. The optical density at wavelengths 590 nm and 750 nm were measured daily for 7 days. Based on these measurements, we recorded the Average Well Color Development (AWCD) for each well, calculated as the sum of (OD_590-750_) for all 31 carbon sources in the ECO plate. Additionally, we determined the Single Carbon Source Metabolism Ratio, which is calculated as (OD_590-750_) of a single carbon source multiplied by 100% divided by the sum of (OD_590-750_) of each carbon source.

Glutamate dehydrogenase (GDH), glutamine oxoglutarate aminotransferase (GOGAT), and glutamine synthetase (GS) are three key enzymes that play significant roles in nitrogen metabolism. GDH is closely associated with the utilization of nitrogen sources (Spinelli et al., 2017), GOGAT is involved in the accumulation of nitrogen sources and GS is responsible for the reuse of nitrogen sources (Hassan et al., 2020). To determine the nitrogen metabolism, we examined the activity levels of glutamate dehydrogenase (GDH) (BC1460, Solarbio, Beijing, China), glutamine oxoglutarate aminotransferase (GOGAT) (BC0070, Solarbio, Beijing, China), and glutamine synthetase (GS) (BC0910, Solarbio, Beijing, China) (Wen et al., 2021).

To determine the intracellular ATP levels, an Enhanced ATP assay kit was used according to the instructions (S0027, Beyotime).

### Real-time quantitative reverse transcription-PCR (RT-qPCR)

RNA isolation, cDNA synthesis, and PCR amplification were performed as described previously (Wang et al., 2023). The bacterial strains were initially grown overnight in LB broth and then diluted 1:100 into 5 mL LB broth supplemented with the antibiotics. After being cultured for 8 h, the cells were harvested for total RNA extraction using the RNAprep pure bacteria kit (DP430, TIANGEN, Beijing, China). To perform the reverse transcription, 400 ng of the extracted RNA was used, following the HiScript III RT SuperMix protocol for qPCR (with gDNA wiper) (R123-01, Vazyme, Nanjing, China). Subsequently, 1 μL of the synthesized cDNA was used for RT-qPCR (Q331-02, Vazyme, Nanjing, China), and RT-qPCR was performed by the QuantStudio 3 PCR system (Thermofisher, USA). The primers were synthesized by Shanghai Sangon Biotechnology Company (Shanghai, China), which were illustrated in the Supplementary Table S1. The internal control 16S rRNA was used to normalize the gene expression. The relative gene expression levels were determined using the 2^-ΔΔCT^ method (Wang et al., 2023).

### Conjugation experiment

Coculture experiments were performed according to Tran et al. (2022). The recipient strain AB43-RR and the donor strain Aby1 that carries the *bla_oxa-23_* gene were, respectively, inoculated in LB broth. Subsequently, bacterial suspensions were prepared for each strain, ensuring a turbidity equivalent to an OD_600_ = 0.5. The Aby1 and AB43-RR were mixed and cultured overnight in LB broth at 37°C (Bi et al., 2018). To select transconjugants, we plated the cultures onto LB agar plates supplemented with RIF (200 μg/mL) and meropenem (4 μg/mL). Randomly selected colonies were subjected to colony PCR to confirm the presence of both the CRISPR-Cas gene and the *bla_oxa-23_* gene in the transformed cells. Transformants that exhibited resistance to carbapenems and harbored the *bla_oxa-23_* gene were identified as transconjugants. The conjugation frequency was determined by calculating the ratio of transconjugants to donor cells (Liu et al., 2020).

### Untargeted metabolomics analysis using UHPLC

To detect the metabolomics of the AB43 and AB43ΔCRISPR-Cas at 0 and 1/4 MIC, we employed a Vanquish UHPLC System (Thermo Fisher Scientific, USA) coupled with an Orbitrap Exploris 120 (Thermo Fisher Scientific, USA) for LC–MS/MS analysis according to Wen et al. (2021). Briefly, the bacteria were grown overnight, washed, snap frozen in liquid nitrogen for 15 min, and stored at −80°C for UPHPLC later.

After thawing, the bacteria were resuspended in 1 mL 50% methanol/water, and were frozen (in liquid nitrogen) and thawed (room temperature) three times, with homogenization in between for 2 min. The lysates were centrifuged at 12,000 rpm for 10 min at 4°C, and the supernatant was removed and vacuum-centrifuge dried. To dissolve the extracts, 300 μL of 2-Amino-3-(2-chloro-phenyl)-propionic acid (4 ppm) solution prepared with 50% methanol/water was added. The solution was filtered using 0.22 μm membrane before LC–MS detection.

For LC-ESI (+)-MS analysis, the mobile phases consisted of B2 (0.1% formic acid in acetonitrile) and A2 (0.1% formic acid in water). Separation was conducted under the following gradient: 0–1 min, 8% B2; 1–8 min, 8–98% B2; 8–10 min, 98% B2; 10–10.1 min, 98–8% B2; 10.1–12 min, 8% B2. For LC-ESI (−)-MS analysis, the mobile phases consisted of B3 (acetonitrile) and A3 (5 mM ammonium formate). Separation was conducted under the following gradient: 0–1 min, 8% B3; 1–8 min, 8–98% B3; 8–10 min, 98% B3; 10–10.1 min, 98–8% B3; 10.1–12 min, 8% B3. Mass spectrometric detection of metabolites was performed on Orbitrap Exploris 120 (Thermo Fisher Scientific, USA) with an ESI ion source.

The raw MS data (wiff. Scan files) were initially converted to mzXML files using Proteowizard (v3.0.8789) MSConvert. These mzXML files were then imported into the R XCMS software for subsequent data processing, including alignment, peak detection, and retention-time corrections. To perform multivariate statistical analysis and principal component analysis (PCA), we utilized SIMCA software (version 14.0). The heat maps were generated using R. Furthermore, to gain insights into the functional annotation of metabolites and perform enrichment analysis of metabolic pathways, we relied on the database provided by the Kyoto Encyclopedia of Genes and Genomes (KEGG).

### Statistical analyses

Statistical analysis was performed by SPSS software. All data are presented as the mean ± SD. Unless otherwise noted, an unpaired t-test between two groups or one-way ANOVA between multiple groups was used to calculate *p*-values (*p < 0.05; **p < 0.01; ***p < 0.001).

## Results

### Sub-MIC increases the fitness cost of the CRISPR-Cas system

**Figure 1:**
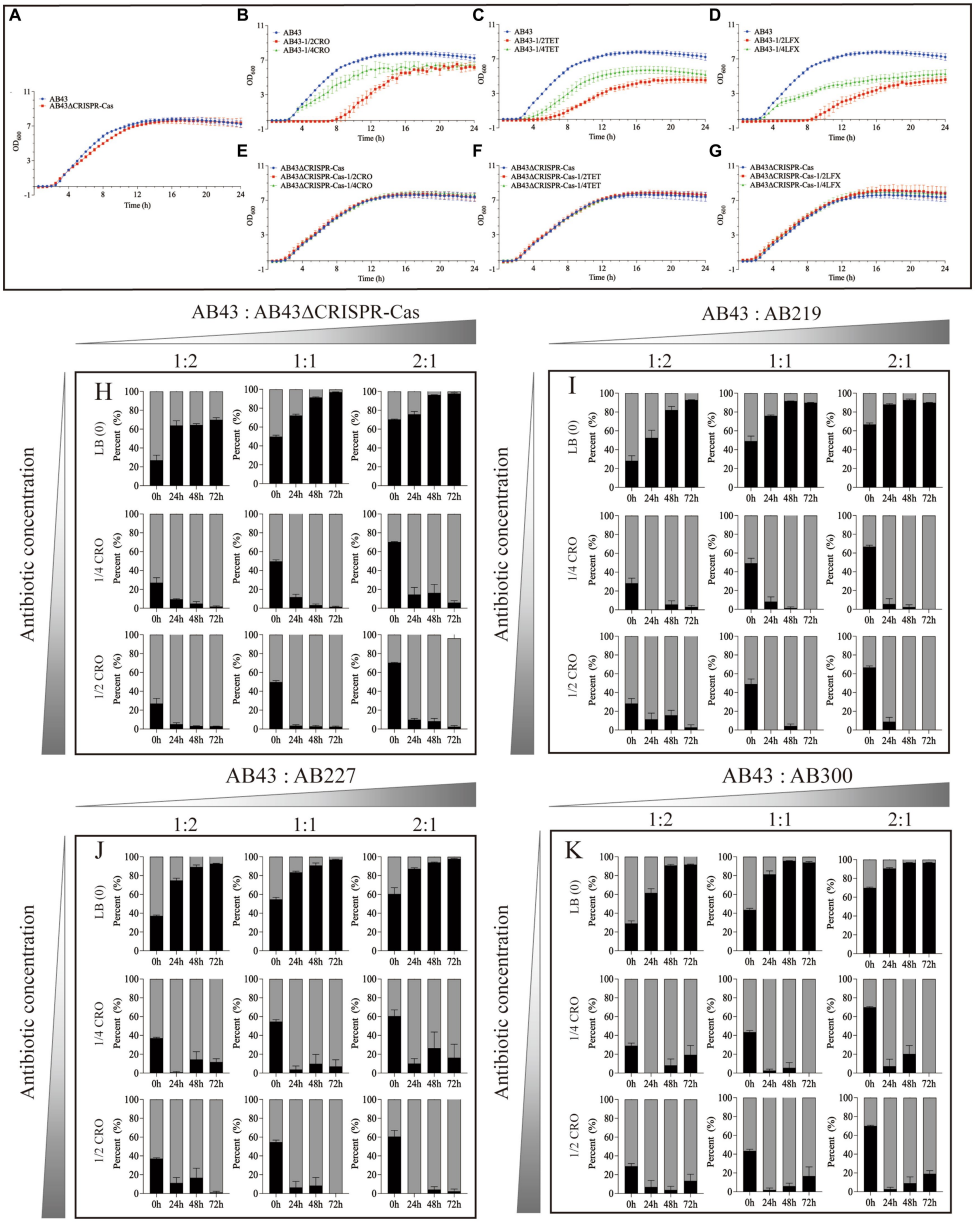
The growth curves **(A–G)** and *in vitro* competition experiments **(H–K)** under sub-MIC (*n* = 3, mean ± SD). The growth curve of AB43 strain was determined in LB broth **(A)** without antibiotics, **(B)** with 1/4 and 1/2 MIC CRO, **(C)** 1/4 and 1/2 MIC TET, and **(D)** 1/4 and 1/2 MIC LFX. The growth curve of AB43ΔCRISPR-Cas strain was determined in LB broth **(A)** without antibiotics, **(E)** with 1/4 and 1/2 MIC CRO, **(F)** 1/4 and 1/2 MIC TET, and **(G)** 1/4 and 1/2 MIC LFX. *In vitro* competition experiments of **(H)** AB43 and AB43ΔCRISPR-Cas, **(I)** AB43 and AB219, **(J)** AB43 and AB227, and **(K)** AB43 and AB300 under 0, 1/4 MIC, 1/2 MIC CRO at different ratios of 1:2, 1:1, and 2:1.

We first determined the MICs of the AB43 and AB43ΔCRISPR-Cas. As shown in Supplementary Table S2, the MICs of CRO were 16 mg/L and 512 mg/L, LFX were 0.25 mg/L and 64 mg/L, and TET were 0.5 mg/L and 512 mg/L, for AB43 and AB43ΔCRISPR-Cas, respectively. We then compared the growth between the two strains at 1/2 and 1/4 sub-MIC (Figure 1; Supplementary Figure S1). Both 1/2 and 1/4 sub-MIC slowed down the growth of AB43 (Figures 1B–D; Supplementary Figures S1B–D), but did not affect the growth of AB43ΔCRISPR-Cas (Figures 1E–G; Supplementary Figures S1E–G).

We next conducted *in vitro* competition experiments. The AB43 and AB43ΔCRISPR-Cas strains were mixed at three different ratios (AB43:AB43ΔCRISPR-Cas = 1:2, 1:1, 2:1) in LB broth with or without antibiotics (0, 1/4 MIC, 1/2 MIC).

In LB broth without antibiotics, the relative abundance of AB43 was rapidly increased from 27.0% ± 9.3% at 0 h to 63.9% ± 8.7% at 24 h, reached 64.5% ± 2.2% at 48 h, and finally reached 69.8% ± 4.0% at 72 h from initial ratios of 1:2 (Figure 1H). In LB broth without antibiotics, the relative abundance of AB43 was rapidly increased from 49.8% ± 3.8% at 0 h to 72.2% ± 3.6% at 24 h, reached 91.5% ± 1.4% at 48 h, and finally reached 97.0% ± 0.6% at 72 h from initial ratios of 1:1 (Figure 1H). In LB broth without antibiotics, the relative abundance of AB43 was rapidly increased from 70.3% ± 0.7% at 0 h to 75.8% ± 5.1% at 24 h, reached 96.3% ± 0.3% at 48 h, and finally reached 97.6% ± 1.4% at 72 h from initial ratios of 2:1 (Figure 1H). Compared with AB43ΔCRISPR-Cas in LB broth without antibiotics, the AB43 exhibited considerable competitiveness regardless of the initial mixed ratio.

In LB broth with 1/4 MIC CRO, the relative abundance of AB43 was rapidly decreased from 27.0% ± 9.3% at 0 h to 9.6% ± 0.8% at 24 h, reached 5.0% ± 3.1% at 48 h, and finally reached 1.7% ± 1.2% at 72 h from initial ratios of 1:2 (Figure 1H). In LB broth with 1/4 MIC CRO, the relative abundance of AB43 was rapidly increased from 49.8% ± 3.82 at 0 h to 11.8% ± 7.2% at 24 h, reached 3.3% ± 2.9% at 48 h, and finally reached 1.6% ± 1.4% at 72 h from initial ratios of 1:1 (Figure 1H). In LB broth with 1/4 MIC CRO, the relative abundance of AB43 was rapidly increased from 70.3% ± 0.7% at 0 h to 14.5% ± 13.0% at 24 h, reached 16.3% ± 15.3% at 48 h, and finally reached 6.0% ± 3.6% at 72 h from initial ratios of 2:1 (Figure 1H).

In LB broth with 1/2 MIC CRO, the relative abundance of AB43 was rapidly decreased from 27.0% ± 9.3% at 0 h to 5.2% ± 2.5% at 24 h, reached 3.2% ± 0.4% at 48 h, and finally reached 2.9% ± 0.4% at 72 h from initial ratios of 1:2 (Figure 1H). In LB broth with 1/2 MIC CRO, the relative abundance of AB43 was rapidly increased from 49.8% ± 3.8% at 0 h to 3.6% ± 2.4% at 24 h, reached 2.8% ± 2.6% at 48 h, and finally reached 2.4% ± 1.6% at 72 h from initial ratios of 1:1 (Figure 1H). In LB broth with 1/2 MIC CRO, the relative abundance of AB43 was rapidly increased from 70.3% ± 0.7% at 0 h to 9.8% ± 2.4% at 24 h, reached 8.3% ± 5.1% at 48 h, and finally reached 2.4% ± 2.1% at 72 h from initial ratios of 2:1 (Figure 1H).

At both 1/4 MIC and 1/2 MIC CRO, compared with AB43, AB43ΔCRISPR-Cas rapidly increased and became the dominant strain (Figure 1H). Similar results between the AB43ΔCRISPR-Cas and AB43 in 1/4 MIC LFX and 1/2 MIC TET were found and shown in Supplementary Figure S2A.

To allow for a more accurate representation of the clinical context, we selected three clinical strains (AB219, AB227, AB300) that did not possess the CRISPR-Cas system but had a similar genetic background to the AB43 strain for *in vitro* competition experiments. Compared with the AB219, AB227, and AB300 in LB broth without antibiotics, the AB43 exhibited competitiveness regardless of the initial mixed ratio (Figures 1I–K), while at 1/4 MIC and 1/2 MIC CRO, the AB43 lost competitiveness regardless of the initial mixed ratio (Figures 1I–K). Similar results of the AB219, AB227, AB300 and AB43 in 1/4 MIC LFX, 1/4 MIC TET, 1/2 MIC LFX, and 1/2 MIC TET were shown in Supplementary Figures S2B–D, S3B–D.

### Sub-MIC inhibits the expression of CRISPR-Cas in *Acinetobacter baumannii*

We next focused on two specific concentrations: 0 and 1/4 MIC in the following experiments. The reason why we chose 1/4 MIC was based on previous studies on residual environmental antibiotic concentrations, which typically fall within the range of 1/4 to 1/230 of the MIC (Chow et al., 2021).

**Figure 2:**
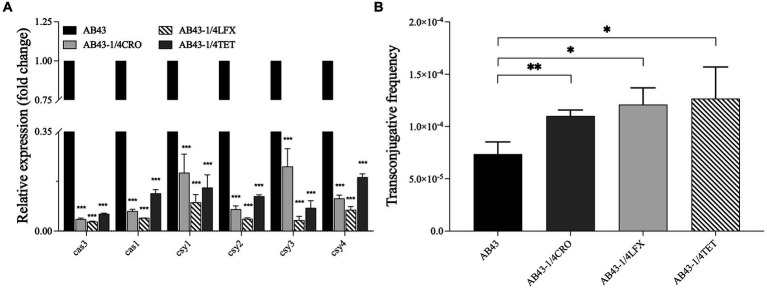
The CRISPR-cas gene expression and the conjugation frequency in AB43 under sub-MIC (*n* = 3, mean ± SD). **(A)** CRISPR-related genes (*cas3*, *cas1*, *csy1*, *csy2*, *csy3*, and *csy4*) expression in AB43 after exposure to antibiotics. **(B)** Conjugation frequency under sub-MIC between AB43 and AB43-RR. *p < 0.05; **p < 0.01, ***p < 0.001, one-way ANOVA.

To examine the sub-MIC effect on the CRISPR-Cas-related gene expression, we carried out RT-qPCR to examine the expression of *cas3*, *cas1*, *csy1*, *csy2*, *csy3*, and *csy4* in AB43. The expression of *cas3*, *cas1*, *csy1*, *csy2*, *csy3*, and *csy4* were mild downregulated in AB43 at sub-MIC (1/4 MIC CRO, 1/4 MIC LFX, and 1/4 MIC TET) (Figure 2A).

To further assess the effectiveness of the CRISPR-Cas system against invading foreign genetic elements, we conducted conjugation experiments. PCR analysis was performed to confirm foreign genetic element *bla_oxa-23_* (Supplementary Figure S4). We found AB43 could transfer plasmids and successfully acquire the *bla_oxa-23_* gene, and we observed a significantly higher conjugation frequency of AB43 at sub-MIC (Figure 2B).

### Metabonomic analysis of the strains at sub-MIC

To investigate the molecular mechanisms by which the CRISPR-Cas system was inhibited under sub-MIC, we performed metabonomic analysis for AB43 and AB43ΔCRISPR-Cas at 0 and 1/4 MIC antibiotic.

As shown in Supplementary Figures S5A,B, our PCA analyses showed that in both positive and negative ion modes, the differences between quality control (QC) samples are minimal, indicating low internal standard variance and high quality of experimental data. After removing the QC samples, further PCA analysis revealed that the AB43 and AB43ΔCRISPR-Cas groups exhibited intra-group clustering and inter-group separation trends in positive and negative ion modes, indicating metabolic differences between AB43 and AB43ΔCRISPR-Cas (Supplementary Figures S5C,D).

In AB43, there was an inter-group separation between 0 and 1/4 MIC in both positive and negative ion modes. The AB43 groups at 1/4 MIC were relatively close, suggesting the similar impact of different antibiotics on the metabolic pattern in AB43 (Supplementary Figures S4C,D). On the contrary, in the AB43ΔCRISPR-Cas groups, the inter-group separation trend was not apparent between 0 and 1/4 MIC, indicating a minimal impact of antibiotics on the metabolic pattern in AB43ΔCRISPR-Cas.

A total of 241 and 113 metabolites were identified in positive and negative ion detection modes, respectively, (variable importance in projection (VIP) > 0.05; log_2_ fold change (FC), < −2 or > 2; *p* < 0.05). There were 126 (37 upregulated and 89 downregulated) differential metabolites identified between AB43 and AB43ΔCRISPR-Cas without antibiotics. In addition, 119 (34 upregulated and 85 downregulated) differential metabolites were identified between AB43 and AB43ΔCRISPR-Cas in 1/4 CRO, 111 (35 upregulated and 76 downregulated) differential metabolites were identified between AB43 and AB43ΔCRISPR-Cas in 1/4 LFX, and 113 (27 upregulated and 86 downregulated) differential metabolites were identified between AB43 and AB43ΔCRISPR-Cas in 1/4 TET, respectively.

**Figure 3:**
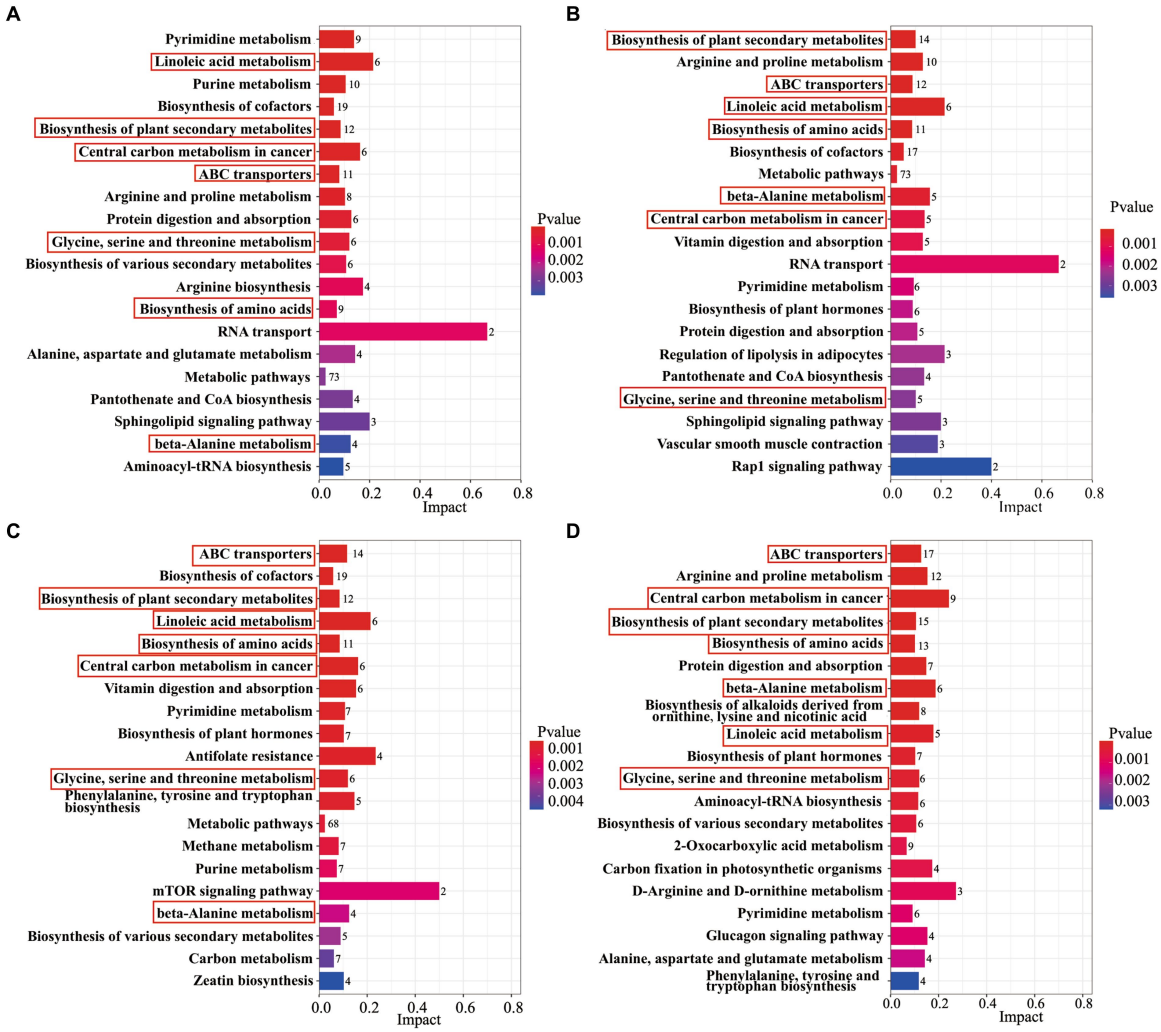
An analysis of KEGG enrichment pathways in AB43 and AB43ΔCRISPR-Cas. KEGG enriched pathways of AB43 *VS* AB43ΔCRISPR-Cas in LB at **(A)** 0, **(B)** 1/4 CRO, **(C)** 1/4 LFX, and **(D)** 1/4 TET. Seven shared metabolic pathways were outlined in red rectangles.

The differential metabolites were annotated by the KEGG database for biochemical metabolic and signal transduction pathways. The results showed that 7 metabolic pathways were changed between AB43 and AB43ΔCRISPR-Cas under 0, 1/4 MIC CRO, 1/4 MIC LFX, and 1/4 MIC TET (Figure 3), including Linoleic acid metabolism, Biosynthesis of plant secondary metabolites, Central carbon metabolism in cancer, ABC transporters, Glycine, serine and threonine metabolism, Biosynthesis of amino acids, and beta-Alanine metabolism.

Among the shared differential metabolites in 7 metabolic pathways, we found the levels of 16 metabolites were significantly altered (Table 1). Interestingly, the level of Sphinganine was upregulated in AB43 compared to AB43ΔCRISPR-Cas without antibiotics, but was downregulated in AB43 compared to AB43ΔCRISPR-Cas at 1/4 MIC (Table 1). No significant differences in Sphinganine levels in AB43ΔCRISPR-Cas were found under different antibiotic pressures. In addition, Sphinganine was significantly downregulated at 1/4 MIC compared to 0 in AB43. (average Log_2_FC = −3.00 in 1/4 CRO, average Log_2_FC = −2.52 in 1/4 LFX, and average Log_2_FC = −4.83 in 1/4 TET). This suggests that Sphinganine may be a key metabolite in the response of the CRISPR-Cas system to antibiotic pressure.

**Table 1:** The selected differential metabolites for AB43/AB43ΔCRISPR-Cas in LB at 0 and 1/4 MIC (VIP > 1 and *p* value <0.05).

Name	AB43 vs. AB43ΔCRISPR-Cas		AB43-1/4 CRO vs. AB43ΔCRISPR-Cas-1/4 CRO		AB43-1/4 LFX vs. AB43ΔCRISPR-Cas-1/4 LFX		AB43-1/4 TET vs. AB43ΔCRISPR-Cas-1/4 TET	
	Log_2_FC	Log_10_(P.value)	Log_2_FC	Log_10_(P.value)	Log_2_FC	Log_10_(P.value)	Log_2_FC	Log_10_(P.value)
(2′E,4’Z,8E)-Colneleic acid	−5.61 ↓	4.74	−5.55 ↓	5.25	−5.04 ↓	4.11	−3.79 ↓	1.65
13S-hydroxyoctadecadienoic acid	−2.28 ↓	3.93	−8.41 ↓	5.76	−4.85 ↓	1.88	−8.27 ↓	5.51
16-Hydroxy hexadecanoic acid	−5.87 ↓	4.19	−5.33 ↓	6.19	−6.43 ↓	6.95	−6.46 ↓	5.71
9,10-Dihydroxystearate	−1.31 ↓	2.35	−1.63 ↓	3.93	−1.57 ↓	3.85	−2.57 ↓	4.44
9,10-Epoxyoctadecenoic acid	−2.50 ↓	2.41	−5.97 ↓	5.70	−5.56 ↓	2.42	−6.46 ↓	5.14
D-Octopine	−2.95 ↓	4.52	−2.17 ↓	3.14	−2.56 ↓	5.02	−3.17 ↓	4.05
GDP	−4.56 ↓	3.60	−3.23 ↓	2.2	−2.39 ↓	3.44	−2.73 ↓	4.28
Glutathione	−6.75 ↓	5.59	3.76 ↑	1.54	−1.92 ↓	1.37	−5.48 ↓	3.68
Glyceric acid	1.53 ↑	3.32	2.04 ↑	4.34	1.76 ↑	3.74	1.70 ↑	3.48
L-Arabinose	2.62 ↑	1.98	2.36 ↑	4.06	3.72 ↑	1.90	2.16 ↑	1.60
L-Arginine	−2.49 ↓	4.07	−2.56 ↓	4.56	−2.10 ↓	1.48	−2.90 ↓	3.43
Lumichrome	−7.30 ↓	4.12	−8.37 ↓	6.74	−5.71 ↓	4.41	−6.12 ↓	4.84
Myristic acid	−3.30 ↓	5.15	−2.97 ↓	1.96	−2.85 ↓	3.44	−2.60 ↓	2.78
Neocembrene	−4.20 ↓	6.31	−2.70 ↓	6.81	−2.45 ↓	5.52	−2.35 ↓	2.79
Sphinganine	1.10 ↑	1.45	−3.03 ↓	3.27	−2.20 ↓	2.46	−4.43 ↓	2.66
UDP	−5.82 ↓	3.67	−3.03 ↓	1.62	−5.85 ↓	4.14	−6.45 ↓	3.86

### CRISPR-Cas impacts *Acinetobacter baumannii* carbon, nitrogen, and energy metabolism by targeting efflux pumps at sub-MIC

Because the central carbon metabolism in cancer, serine–threonine metabolism, Biosynthesis of amino acids, and β-Alanine metabolism pathways are associated with the consumption of carbon, nitrogen, and intracellular ATP levels, we next examined the carbon, nitrogen, and energy metabolism in AB43 and AB43ΔCRISPR-Cas at 0 and 1/4 MIC.

**Figure 4:**
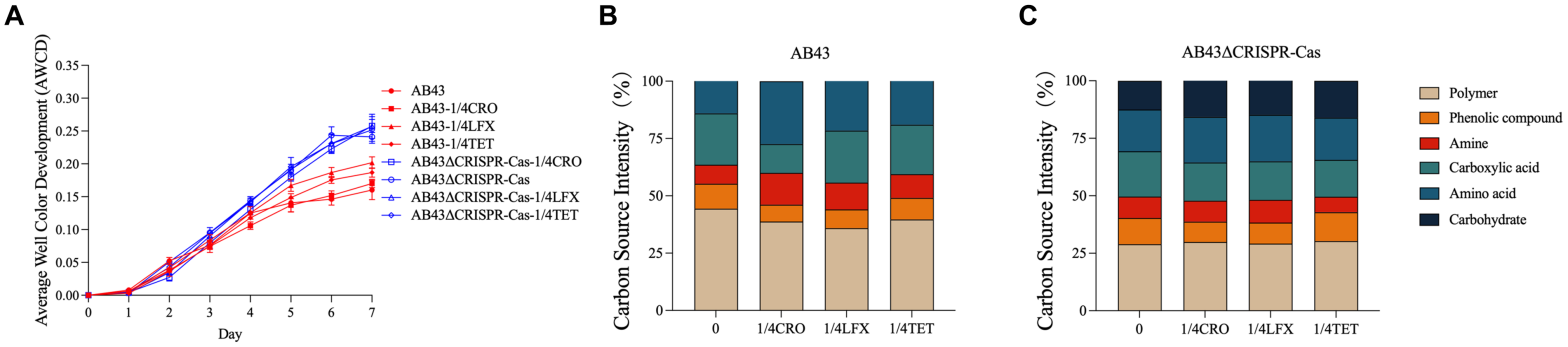
Carbon metabolism for AB43 and AB43ΔCRISPR-Cas at 0 and 1/4 MIC (*n* = 3, mean ± SD). **(A)** Changes of AWCD in AB43 and AB43ΔCRISPR-Cas at 0 and 1/4 MIC. **(B)** The utilization rates of 6 types of carbon sources for AB43 at 0 and 1/4 MIC. **(C)** The utilization rates of 6 types of carbon sources for AB43ΔCRISPR-Cas at 0 and 1/4 MIC.

As shown in Figure 4A, both AB43 and AB43ΔCRISPR-Cas at 0 and 1/4 MIC exhibited a growth lag on the first day followed by an increase in AWCD that is routinely used for measuring the carbon source metabolism capability of microorganisms (Garland and Mills, 1991). On the second day, there was no significant difference in the AWCD between AB43 and AB43ΔCRISPR-Cas. However, AB43ΔCRISPR-Cas subsequently exhibited a higher AWCD than AB43, indicating a higher carbon source consumption rate in AB43ΔCRISPR-Cas. Importantly, compared to the antibiotic-free condition, at 1/4 MIC, AB43 showed an increased average carbon source metabolism rate at 1/4 MIC AB43ΔCRISPR-Cas showed no significant change. This suggests that AB43 requires more carbon sources under antibiotic conditions.

Among the six categorized carbon substrates, the most utilized carbon sources for AB43 were polymer, carboxylic acid, and amino acid. Interestingly, AB43 does not utilize carbohydrates as carbon source (Figure 4B). Interestingly, the most utilized carbon source for AB43ΔCRISPR-Cas were polymer, carboxylic acid, amino acid, and carbohydrate (Figure 4C), highlighting the differential carbon sources between the two strains.

**Figure 5:**
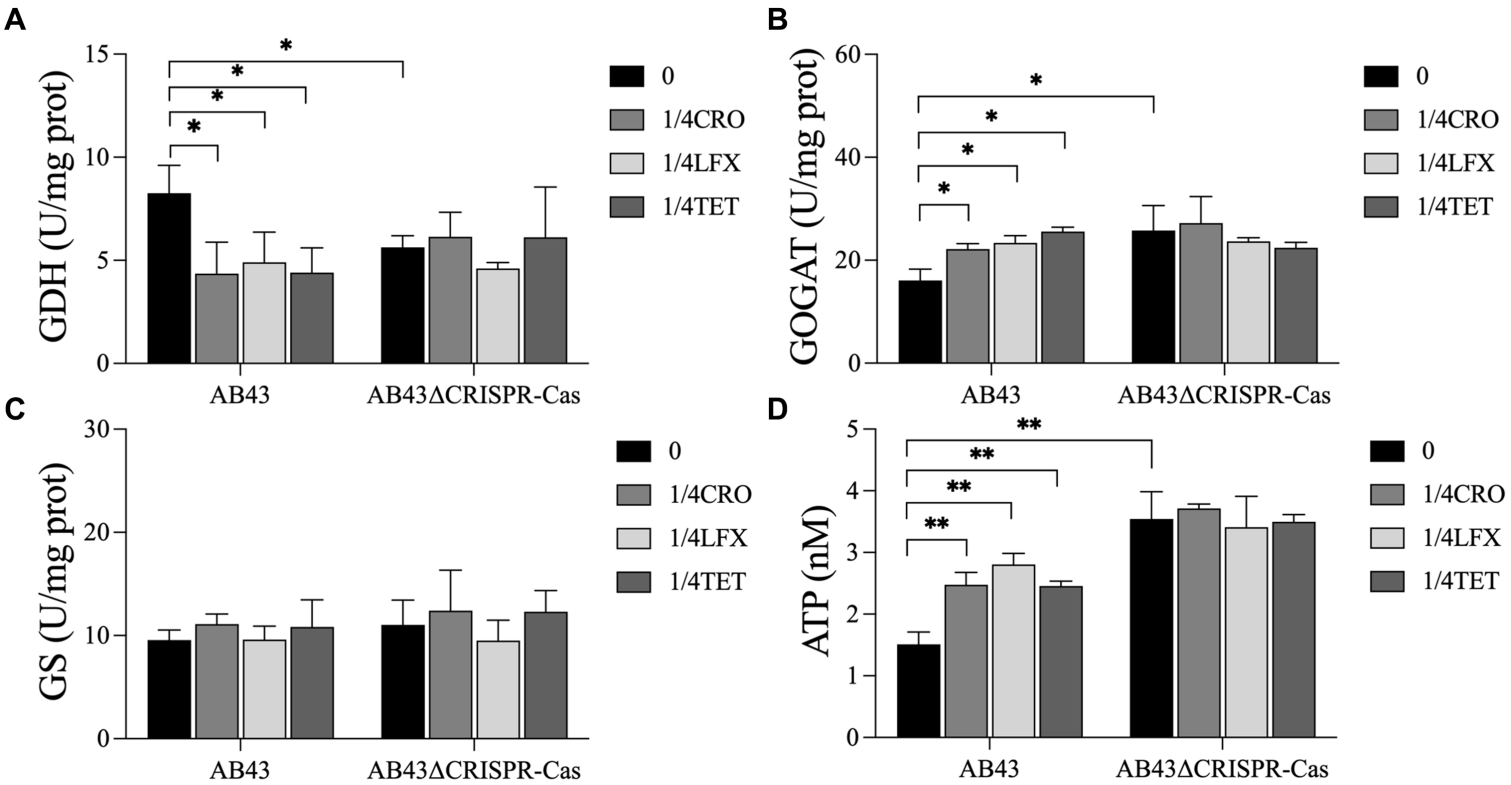
Nitrogen and energy metabolism for AB43 and AB43ΔCRISPR-Cas at 0 and 1/4 MIC (*n* = 3, mean ± SD). The metabolism of nitrogen was evaluated by **(A)** GDH, **(B)** GOGAT and **(C)** GS activity, and energy metabolism was evaluated by **(D)** intracellular ATP. *p < 0.05; **p < 0.01, one-way ANOVA.

We next examined the nitrogen metabolism by assessing the activities of GDH, GOGAT, and GS. Compared to AB43ΔCRISPR-Cas, the enzyme activity of GDH in AB43 was higher while the enzyme activity of GOGAT was lower (Figures 5A–C), indicating that AB43ΔCRISPR-Cas consumes nitrogen sources more rapidly. Furthermore, when compared to AB43 without antibiotics, the enzyme activities of GDH in AB43 at 1/4 MIC were lower, and the enzyme activities of GOGAT were higher. These results indicate that AB43 consumes more nitrogen sources under sub-MIC antibiotic conditions.

We next assessed the energy metabolism levels by examining the ATPase activities in the strains. Compared to AB43ΔCRISPR-Cas, the intracellular level of ATP in AB43 was lower (Figure 5D), indicating that AB43ΔCRISPR-Cas consumes more ATP energy. Additionally, compared to AB43 without antibiotics, the intracellular level of ATP in AB43 at 1/4 MIC was higher (Figure 5D), which indicates that AB43 consumes more ATP under sub-MIC.

**Figure 6:**
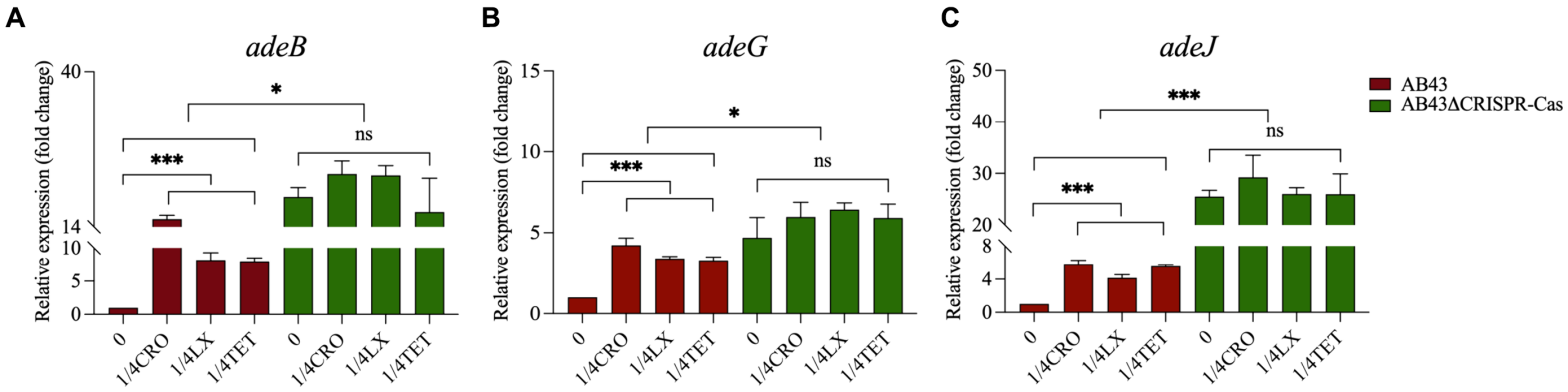
The efflux pump-related gene expression in AB43 and AB43ΔCRISPR-Cas under sub-MIC (*n* = 3, mean ± SD). *p < 0.05; **p < 0.01, one-way ANOVA.

The efflux pump, which utilizes ATP hydrolysis energy, is a defense mechanism for the cell to expel antibiotics and reduce their intracellular concentration under antibiotic pressures. We further examined the expression of efflux pump-related genes in both strains at 0 compared to 1/4 MIC. We found 1/4 MIC significantly upregulated the expression of *adeB*, *adeG*, and *adeJ* in AB43, but not in AB43ΔCRISPR-Cas (Figure 6). Importantly, the gene expression levels in AB43 at 1/4 MIC were lower than those in AB43ΔCRISPR-Cas at either 0 or 1/4 MIC (Figure 6).

## Discussion

Recent studies have indicated a close relationship between the CRISPR-Cas system and bacterial antibiotic resistance (Aydin et al., 2017; Shabbir et al., 2018; Sanderson et al., 2020; Li et al., 2021; Saha et al., 2023). In our previous investigation (Wang et al., 2022), we found that only 2/245 clinical isolates of *A. baumannii* possessed intact CRISPR-Cas systems, and these strains were sensitive to antibiotics. The majority of the isolates had structurally incomplete or missing CRISPR-Cas systems, and their resistance increased with the severity of the system’s deficiency (Wang et al., 2022). The negative strains were mostly multidrug-resistant (Wang et al., 2022). These findings suggest that the inhibition and loss of the CRISPR-Cas system are important factors associated with the development of antibiotic resistance in *A. baumannii*. In our research, we observed that the I-Fb CRISPR-Cas system in *A. baumannii* demonstrated a significant competitive advantage in the absence of antibiotics. However, the bacteria without the CRISPR-Cas system lost its competitive advantage under sub-MIC. The expression of CRISPR-related genes was suppressed, and the conjugation frequency was increased in AB43 under sub-MIC conditions. Further investigations revealed that under sub-MIC conditions, the CRISPR-Cas system primarily affected energy metabolism pathways and increased the demand for nutrients required for growth.

Previous studies (Vale et al., 1812; Westra et al., 2015; Mortensen et al., 2021; Shivram et al., 2021) have demonstrated that the presence of the CRISPR-Cas system leads to fitness costs and affects its frequency and stability within bacterial populations. Different bacteria respond differently to fitness costs under various environmental conditions (Ward et al., 2009; Deris et al., 2013). For example, in *Pseudomonas aeruginosa*, Westra et al. (2015) found that the knockout of a single *csy3* gene has minimal impact on competitive ability, but the deletion of the I-F CRISPR-Cas cluster results in the emergence of fitness costs. This indicates that the fitness cost of the CRISPR-Cas system in *Pseudomonas aeruginosa* has a relatively low fitness cost in the absence of bacteriophage exposure, which can bring more benefits to bacteria against foreign nucleic acids. However, Vale et al. (1812) found that the expression of cas9 or csn2 incurs significant fitness costs in the II-A CRISPR-Cas system in *Streptococcus thermophilus*. In our study, we found that the I-Fb CRISPR-Cas system in *A. baumannii* had relatively low fitness costs in the absence of antibiotic pressure and exhibited significant competitive advantages, indicating the presence of fitness costs associated with the CRISPR-Cas system increased under sub-MIC conditions. This is consistent with Westra et al. (2015), but contradict the findings of Vale et al. (1812). It is worth noting that *A. baumannii* and *Pseudomonas aeruginosa* are the same type I-F CRISPR-Cas system, whereas *Streptococcus thermophilus* possesses a type II-A system. These variations in CRISPR-Cas systems could potentially explain the differences in outcomes between the different studies.

In addition, host regulatory factors modulate the operation of the CRISPR-Cas system to reduce fitness costs and maintain its functionality (Shivram et al., 2021). Studies have shown that external signals such as glucose levels (Patterson et al., 2015), iron levels (Ahator et al., 2020), and extracellular stress (Perez-Rodriguez et al., 2011) can control the CRISPR-Cas system through various pathways, thereby regulating fitness costs. For example, Lin et al. (2016) have found that imipenem can inhibit the acquisition of DNA by the CRISPR-Cas system in *Klebsiella pneumoniae*, thereby reducing the activity of the I-E type CRISPR-Cas system. Additionally, Ding et al. (2022) has shown that sub-MIC concentrations may promote plasmid transfer in *Escherichia coli* and β-lactam antibiotics can facilitate plasmid transfer in *Staphylococcus aureus* (Barr et al., 1986). These findings are consistent with our research, indicating that AB43 was more prone to acquiring exogenous antibiotic resistance genes under sub-MIC conditions. Additionally, we observed the expressions of CRISPR-Cas related genes were decreased in AB43 under sub-MIC conditions, indicating a reduced capacity to suppress exogenous antibiotic-resistance genes in AB43 under sub-MIC conditions.

Previous studies have demonstrated that antibiotics disrupt the metabolic state and respiratory function of bacteria, affecting downstream metabolic processes (Stokes et al., 2019; Lobritz et al., 2022). Antibiotic treatment has been shown to induce oxidative stress responses in bacteria (Foti et al., 2012). This oxidative stress is associated with metabolic remodeling, characterized by an increase in the abundance of central carbon metabolites (Belenky et al., 2015). Similar changes in central carbon metabolism have also been observed by Yaeger et al. (2023) in their study on *E. coli* treated with sub-MIC concentrations of antibiotics, aligning with our findings. Our metabolomics analysis revealed alterations in central carbon metabolism in *A. baumannii* at sub-MIC, with increased carbon source consumption rates observed in AB43 at sub-MIC. In addition to carbon sources, nitrogen sources are essential for bacterial viability. Studies have identified GDH, GOGAT, and GS as key enzymes in nitrogen metabolism (Spinelli et al., 2017; Hassan et al., 2020). GDH catalyzes the conversion of glutamate, and the enzyme activity of GDH is positively correlated with nitrogen utilization (Spinelli et al., 2017). GOGAT catalyzes the production of glutamine from glutamate, and its activity is significantly correlated with nitrogen accumulation (Hassan et al., 2020). In our study, we observed the enzyme activities of GDH decreased and the enzyme activity of GOGAT was increased in *A. baumannii* under sub-MIC, indicating increased nitrogen source consumption under sub-MIC conditions. This could be a reason why the AB43∆CRISPR-Cas strain lacks a competitive advantage in the absence of antibiotics since it requires more energy to sustain life activities.

Bacteria can generate ATP through central carbon metabolism, providing the primary and direct energy supply for life activities (Verschueren et al., 2019). The efflux pumps are an energy-consuming self-protective mechanism that has evolved over a long period of time and allows harmful substances within the cell to be expelled, thereby preventing the accumulation of toxic compounds. Under antibiotic pressure, the efflux pumps are one of the most rapid and effective defense mechanisms employed by bacteria. However, overexpression of efflux pumps consumes significant energy and affects the fitness cost of bacteria (Lin et al., 2019). This is consistent with our research and the expression of efflux pump-related genes and the intracellular levels of ATP were significantly increased in AB43 at sub-MIC. Wang et al. (2022) also revealed the role of the CRISPR-Cas system in modulating the activity of efflux pumps. Our results indicated that the CRISPR-Cas system in AB43 was inhibited under sub-MIC conditions, which might lead to a dependence on efflux pumps for combating exogenous antibiotics in *A. baumannii*. However, this defense mechanism required a higher energy expenditure compared to normal conditions, resulting in an increased fitness cost for the bacteria.

## Conclusion

To conclude, we found a significant competitive advantage of the I-Fb CRISPR-Cas system in *A. baumannii* without antibiotics. However, the fitness cost of the CRISPR-Cas system in *A. baumannii* was increased under sub-MIC conditions, leading to the loss of competitive advantage. Additionally, our findings indicated sub-MIC affects carbon, nitrogen, energy metabolism, and efflux pumps in *A. baumannii*. Our research findings suggest that sub-MIC may be one of the potential factors leading to the loss of the CRISPR-Cas system in *A. baumannii*.

## Data availability statement

The original contributions presented in the study are publicly available. This data can be found at: https://www.ebi.ac.uk/metabolights/editor/study/MTBLS9524.

## Author contributions

TY: Data curation, Methodology, Supervision, Validation, Writing – original draft, Writing – review & editing. JHu: Methodology, Validation, Writing – review & editing. XH: Methodology, Validation, Writing – review & editing. JHa: Writing – review & editing. PZ: Writing – review & editing. TG: Writing – review & editing. GB: Writing – review & editing. GL: Funding acquisition, Writing – review & editing.

## Footnotes

^1^https://www.ncbi.nlm.nih.gov/nuccore/2095284784

## Supplementary material

SUPPLEMENTARY FIGURE S1 The growth rate of AB43 and AB43ΔCRISPR-Cas strains were determined at sub-MIC concentrations (*n* = 3, mean ± SD). The growth rate of AB43 strains were determined in LB broth **(A)** without antibiotics, **(B)** with 1/4 and 1/2 MIC CRO, **(C)** with 1/4 and 1/2 MIC TET and **(D)** with 1/4 and 1/2 MIC LFX. The growth rate of AB43ΔCRISPR-Cas strain was determined in LB broth **(A)** without antibiotics, **(E)** with 1/4 and 1/2 MIC CRO, **(F)** with 1/4 and 1/2 MIC TET, and **(G)** with 1/4 and 1/2 MIC LFX.

SUPPLEMENTARY FIGURE S2 *In vitro* competition experiments under sub-MIC (*n* = 3, mean ± SD). *In vitro* competition experiments of **(A)** AB43 and AB43ΔCRISPR-Cas, **(B)** AB43 and AB219, **(C)** AB43 and AB227, and **(D)** AB43 and AB300 were determined under 0, 1/4 MIC, 1/2 MIC LFX at different ratios of 1:2, 1:1, and 2:1.

SUPPLEMENTARY FIGURE S3 *In vitro* competition experiments under sub-MIC (*n* = 3, mean ± SD). *In vitro* competition experiments of **(A)** AB43 and AB43ΔCRISPR-Cas, **(B)** AB43 and AB219, **(C)** AB43 and AB227 and **(D)** AB43 and AB300 were determined under 0, 1/4 MIC, 1/2 MIC TET at different ratios of 1:2, 1:1, and 2:1.

SUPPLEMENTARY FIGURE S4 Colony PCR to verify *blaoxa-23* gene transfer to AB43 in conjugation experiments. The transformant colonies were analyzed for CRISPR-related genes (*cas1*, *cas3*, *csy1*, *csy2*, *csy3*, and *csy4*) and *blaoxa-23* using colony PCR. M: marker; 1: the control; 2-4: the 4 different colonies were analyzed for *Cas3* (2318 bp), *Cas1* (966 bp), *Csy1* (1255 bp), *Csy2* (838 bp), *Csy3* (996 bp), *Csy4* (621 bp) and the *blaoxa23* gene (501 bp).

SUPPLEMENTARY FIGURE S5 Principal component analysis (PCA) score plot of overall samples in both positive and negative ion detection mode. PCA score plot detected overall samples and QC samples in both **(A)** positive and **(B)** negative ion detection mode. PCA score plot was used to further analyze the samples after removing the QC samples in both **(C)** positive and **(D)** negative ion detection mode.

SUPPLEMENTARY TABLE S1 Primer sequences.

SUPPLEMENTARY TABLE S2 MIC of strains.
