# Supplementary figures and images for: Sub-MIC antibiotics increased the fitness cost of CRISPR-Cas in Acinetobacter baumannii
Source: Front Microbiol. 2024 Jul 1;15:1381749. doi: 10.3389/fmicb.2024.1381749 (PMC11246858; doi:10.3389/fmicb.2024.1381749)

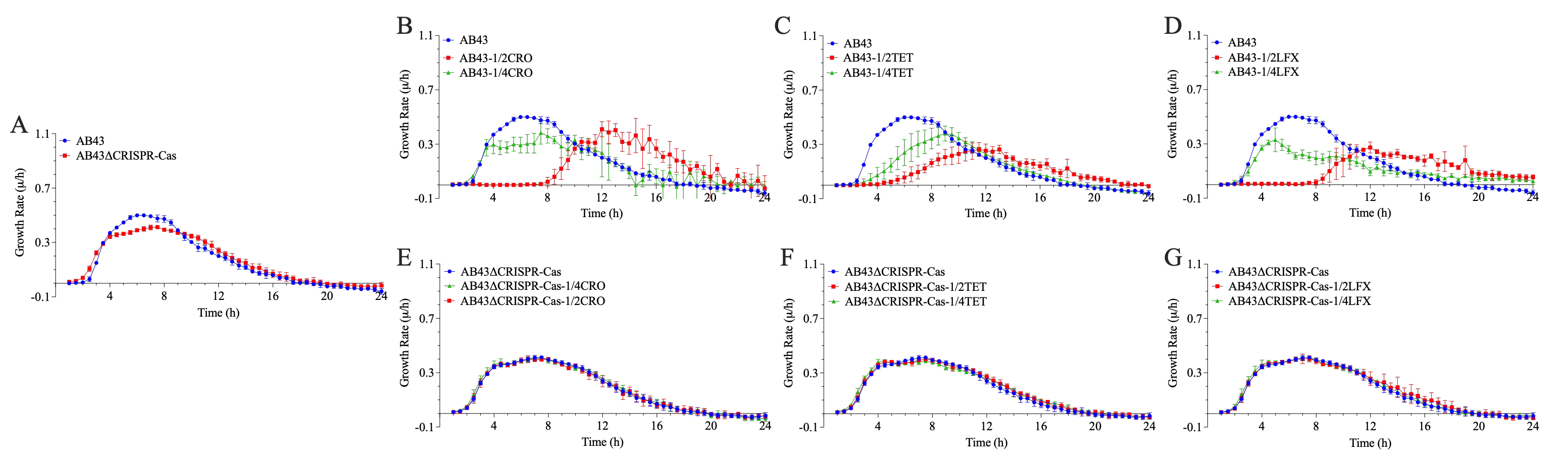

Supplement: SUPPLEMENTARY FIGURE S1 — The growth rate of AB43 and AB43ΔCRISPR-Cas strains were determined at sub-MIC concentrations (n = 3, mean ± SD). The growth rate of AB43 strains were determined in LB broth (A) without antibiotics, (B) with 1/4 and 1/2 MIC CRO, (C) with 1/4 and 1/2 MIC TET and (D) with 1/4 and 1/2 MIC LFX. The growth rate of AB43ΔCRISPR-Cas strain was determined in LB broth (A) without antibiotics, (E) with 1/4 and 1/2 MIC CRO, (F) with 1/4 and 1/2 MIC TET, and (G) with 1/4 and 1/2 MIC LFX. [file Image_1.tiff]

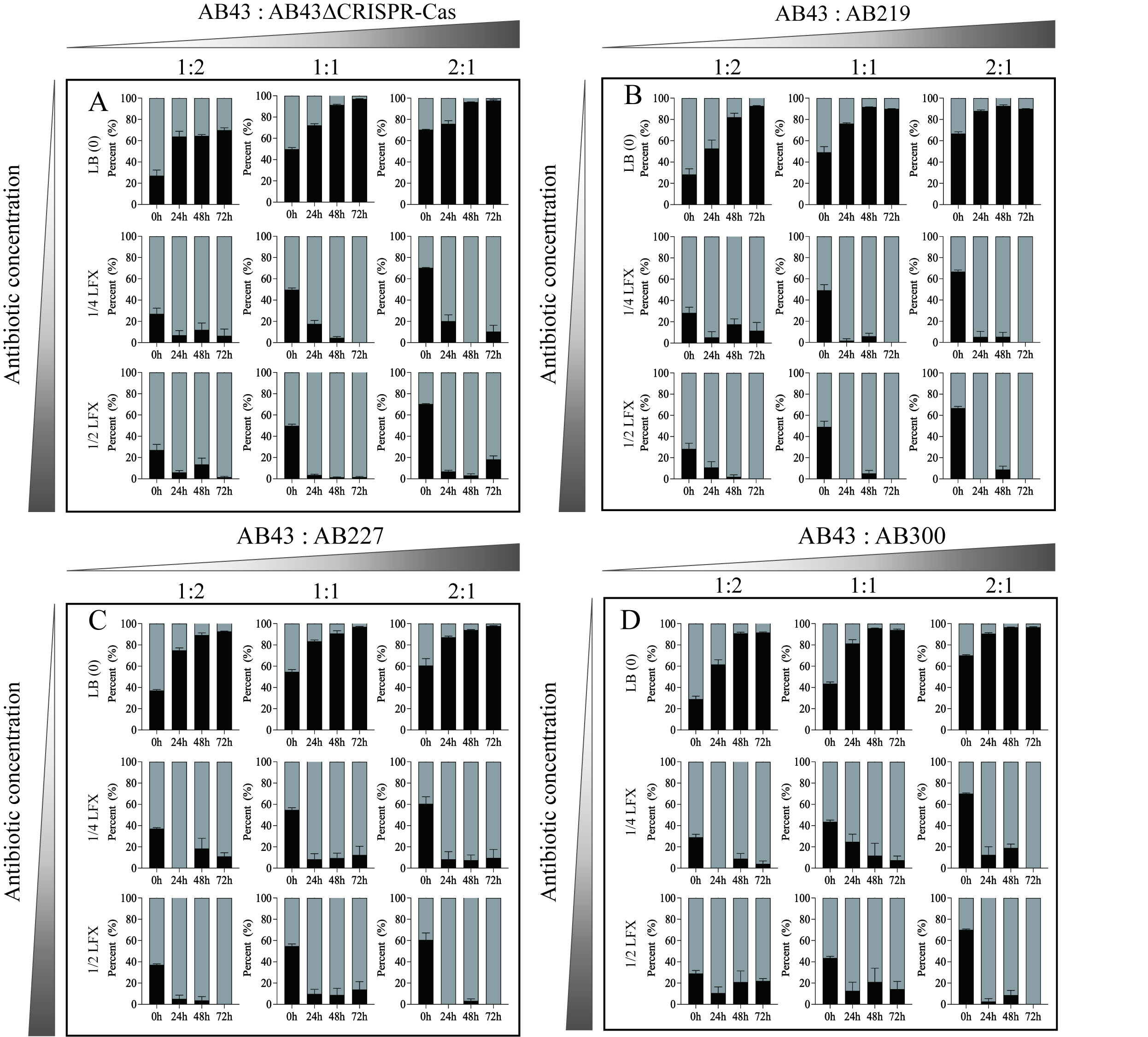

Supplement: SUPPLEMENTARY FIGURE S2 — In vitro competition experiments under sub-MIC (n = 3, mean ± SD). In vitro competition experiments of (A) AB43 and AB43ΔCRISPR-Cas, (B) AB43 and AB219, (C) AB43 and AB227, and (D) AB43 and AB300 were determined under 0, 1/4 MIC, 1/2 MIC LFX at different ratios of 1:2, 1:1, and 2:1. [file Image_2.tif]

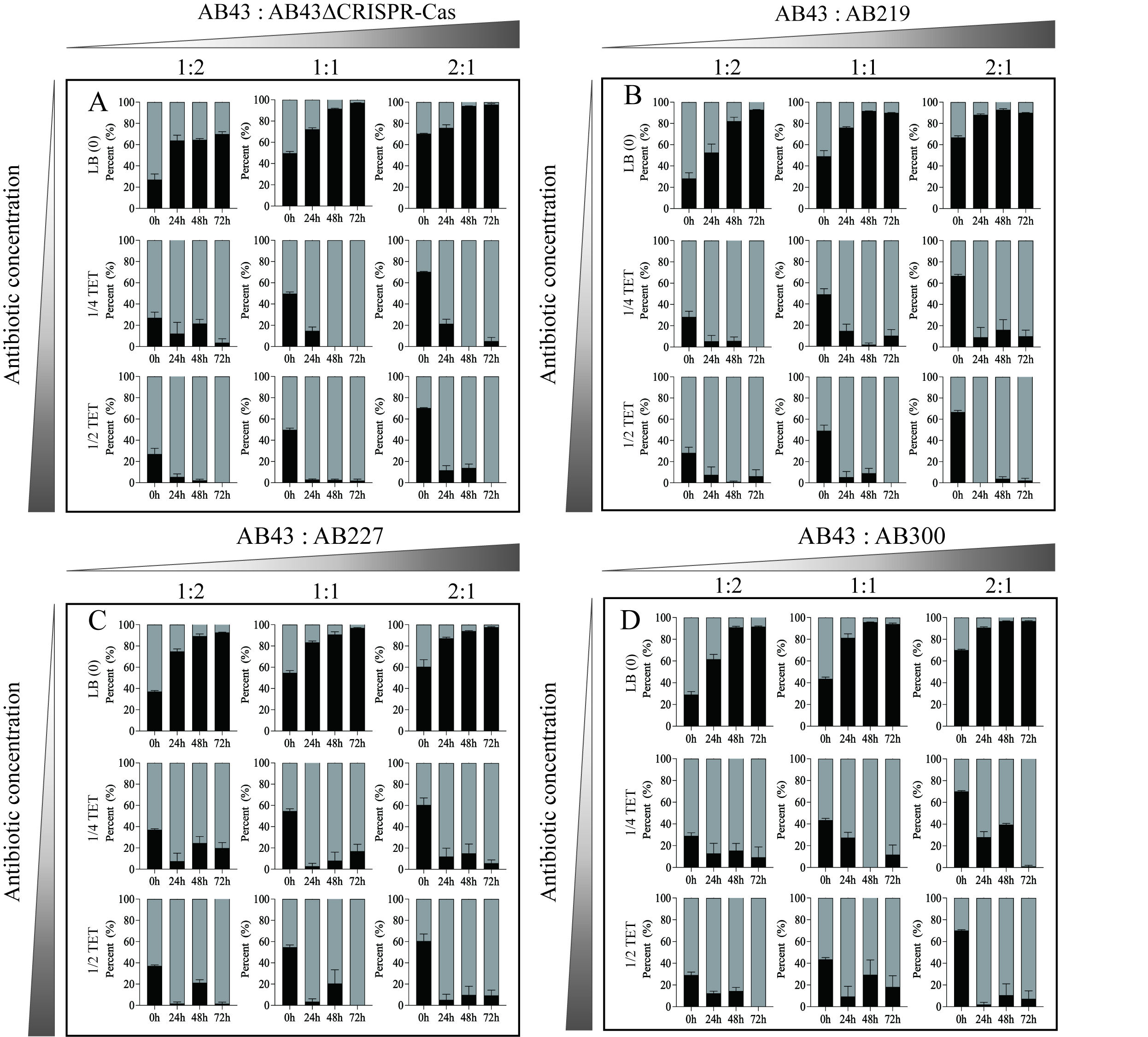

Supplement: SUPPLEMENTARY FIGURE S3 — In vitro competition experiments under sub-MIC (n = 3, mean ± SD). In vitro competition experiments of (A) AB43 and AB43ΔCRISPR-Cas, (B) AB43 and AB219, (C) AB43 and AB227 and (D) AB43 and AB300 were determined under 0, 1/4 MIC, 1/2 MIC TET at different ratios of 1:2, 1:1, and 2:1. [file Image_3.tif]

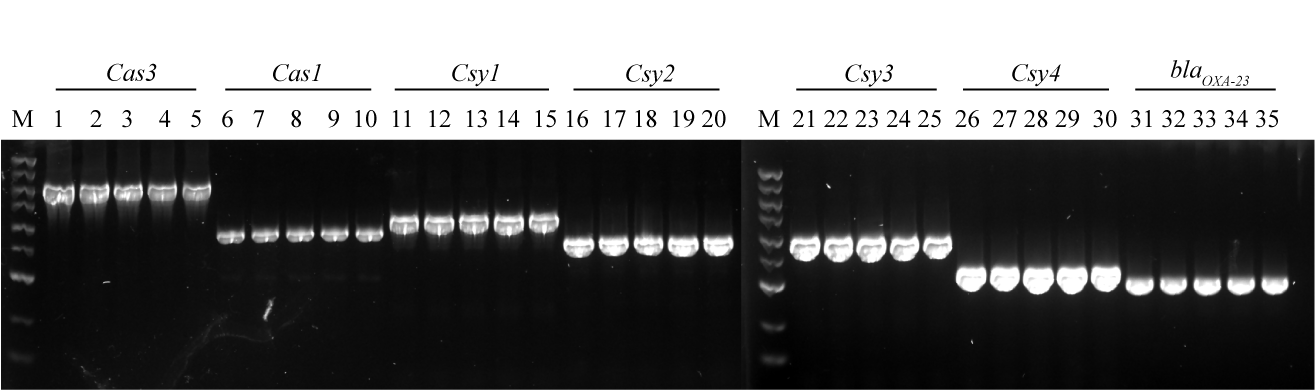

Supplement: SUPPLEMENTARY FIGURE S4 — Colony PCR to verify blaoxa-23 gene transfer to AB43 in conjugation experiments. The transformant colonies were analyzed for CRISPR-related genes (cas1, cas3, csy1, csy2, csy3, and csy4) and blaoxa-23 using colony PCR. M: marker; 1: the control; 2-4: the 4 different colonies were analyzed for Cas3 (2318 bp), Cas1 (966 bp), Csy1 (1255 bp), Csy2 (838 bp), Csy3 (996 bp), Csy4 (621 bp) and the blaoxa23 gene (501 bp). [file Image_4.tif]

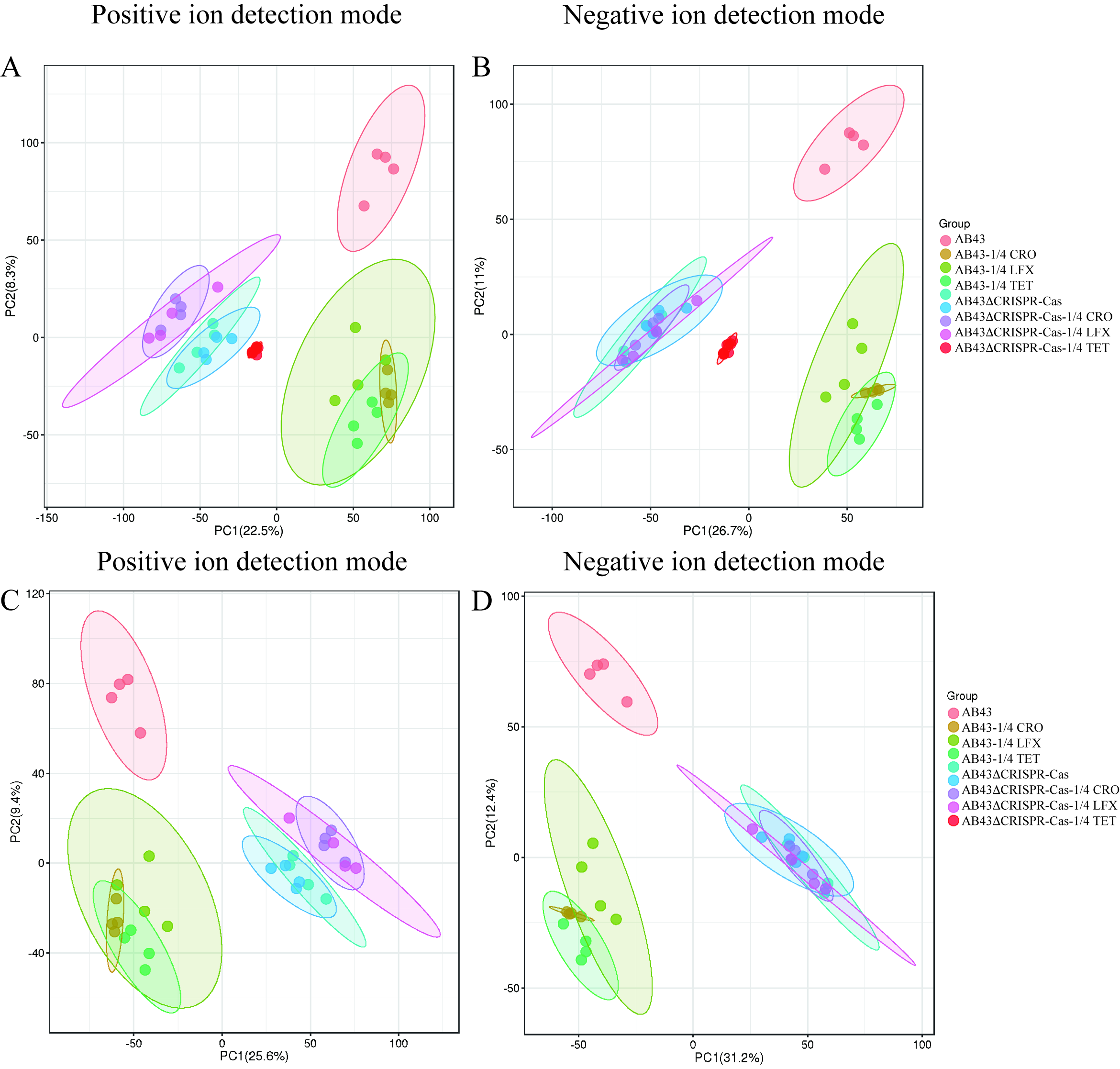

Supplement: SUPPLEMENTARY FIGURE S5 — Principal component analysis (PCA) score plot of overall samples in both positive and negative ion detection mode. PCA score plot detected overall samples and QC samples in both (A) positive and (B) negative ion detection mode. PCA score plot was used to further analyze the samples after removing the QC samples in both (C) positive and (D) negative ion detection mode. [file Image_5.tif]
