# Supplementary material for: Sub-MIC antibiotics increased the fitness cost of CRISPR-Cas in Acinetobacter baumannii
Source: Front Microbiol. 2024 Jul 1;15:1381749. doi: 10.3389/fmicb.2024.1381749 (PMC11246858; doi:10.3389/fmicb.2024.1381749)
Supplement: SUPPLEMENTARY TABLE S1 — Primer sequences. [file Table_1.docx]

**Table S1.** **Primer sequences.**

| Gene | Sequences (5’ to 3’) | Length (bp) |
| --- | --- | --- |
| 16s rRNA-F | GTTGTGGCTTTAGGTTTATTATACG | 94 |
| 16s rRNA-R | AAGTTACTCGACGCAATTCG |  |
| qcas3-F | GCCAAGACTTGATTGCGATTGCC | 137 |
| qcas3-R | TAACGACCAACCGTGCTGATAAGC |  |
| qcas1-F | AGTTTTGAACGGAACCCTGAGCAG | 91 |
| qcas1-R | GTGTCGTCGCACTTAGGCCATAG |  |
| qcsy1-F | GGCCGAAAATGAAAGTGCCATCGC | 83 |
| qcsy1-R | CTGCGTCAGGAAGCCACTCATTC |  |
| qcsy2-F | CAGGGGCGTGATGCTTTAGATGC | 117 |
| qcsy2-R | AAGCCAGCCGTGCGTTTGAC |  |
| qcsy3-F | AGGATGTTGCTGCAATGCACTCTC | 117 |
| qcsy3-R | CCGCACCGTATGGCTCAATCGG |  |
| qcsy4-F | GTGTTTGCCAGCTCGGAAAATGAC | 117 |
| qcsy4-R | TGTAATCTTGGCTCGAGGCACTTC |  |
| OXA-23-F | GATCGGATTGGAGAACCAGAR | 501 |
| OXA-23-R | ATTTCTGACCGCATTTCCAT |  |
| Cas3-F | CATCGTGTGAGGCTGATA | 2318 |
| Cas3-R | AGGCAACATGATTTCCCC |  |
| Cas1-F | ATGGAACAACTTAACCCATC | 966 |
| Cas1-R | TCATCTTTCACTCTCCAA |  |
| Csy1-F | ATGGCTGAAAGTATCCATAC | 1255 |
| Csy1-R | CGCATTAAAAAAACTCCTTTGC |  |
| Csy2-F | ATGCGTCATTTTTTATTGATTCCTC | 838 |
| Csy2-R | CAACATGATATTGCCATAACAG |  |
| Csy3-F | GCAGTTGCAAGTGTACTTG | 996 |
| Csy3-R | TTACTCCTTACCACTTTGAC |  |
| Csy4-F | GGAGTAATGATGAATTGGTATC | 621 |
| Csy4-R | TCAAAACTCTGGGACTGTTGT |  |
| qadeB-F | CTTGCATTTACCTGTGGTGT | 169 |
| qadeB-R | GCTTTTCTACTGCACCCAGA |  |
| qadeG-F | GTTGCCGTATTCTTCGGCA | 91 |
| qadeG-R | TGCTGTTGAGGGTACGAATCA |  |
| qadeJ-F | TCATGACCACCCTTGCCT | 113 |
| qadeJ-R | GCTGAGTACGCCACCAAGTA |  |
