# Supplementary material for: Sub-MIC antibiotics increased the fitness cost of CRISPR-Cas in Acinetobacter baumannii
Source: Front Microbiol. 2024 Jul 1;15:1381749. doi: 10.3389/fmicb.2024.1381749 (PMC11246858; doi:10.3389/fmicb.2024.1381749)
Supplement: SUPPLEMENTARY TABLE S2 — MIC of strains. [file Table_2.docx]

**Table S2.** **MIC of strains**

| Strains | Antibiotics (mg / L) | | |
| --- | --- | --- | --- |
|  | Ceftriaxone | Levofloxacin | Tetracycline |
| AB43 | 16 (S) | 0.25 (S) | 0.5 (S) |
| AB43ΔCRISPR-Cas | 512 (R) | 64 (R) | 512 (R) |
| AB219 | >256 (R) | >64 (R) | >128 (R) |
| AB227 | >256 (R) | >64 (R) | >128 (R) |
| AB300 | >256 (R) | 64 (R) | >128 (R) |
